# Intestine epithelial cell-derived extracellular vesicles alleviate inflammation induced by *Clostridioides difficile* TcdB through the activity of TGF-β1

**DOI:** 10.1007/s13273-022-00280-8

**Published:** 2022-07-30

**Authors:** Shuangshuang Wan, Guangzhong Song, Hui Hu, Yaqing Xu, Peng Zeng, Shan Lin, Jun Yang, Jinqin Jiang, Xiaojun Song, Yongneng Luo, Dazhi Jin

**Affiliations:** 1 School of Laboratory Medicine and Bioengineering, Hangzhou Medical College, No. 481 Binwen Rd., Hangzhou, 310053 Zhejiang China; 2 Centre of Laboratory Medicine, People’s Hospital of Hangzhou Medical College, Zhejiang Provincial People’s Hospital, Hangzhou, 310014 Zhejiang China; 3 Key Laboratory of Biomarkers and In Vitro Diagnosis Translation of Zhejiang Province, Hangzhou, 310063 Zhejiang China

**Keywords:** Extracellular vesicles, *Clostridioides difficile*, TGF-β1, TcdB, Regulatory *T* cells, Inflammatory cytokines, Immunotherapy

## Abstract

**Background:**

*Clostridioides difficile* infection (CDI) has been primarily associated with the toxin B (TcdB), one of the three known protein toxins secreted by *C. difficile*, which can activate the intestinal immune system and lead to pathological damage. Even though the biological functions of intestine epithelial cell-derived extracellular vesicles (I-Evs) have been well documented, the role of I-Evs in the process of CDI is still unknown.

**Objectives:**

The protective effect of I-Evs against *C. difficile* TcdB was investigated both in cultured murine colon carcinoma MC38 cells and a mouse model used in this study.

**Results:**

Mouse I-Evs with mean diameter ranging from 100 to 200 nm and a density of 1.09–1.17 g/mL were obtained and confirmed containing the Ev-associated specific surface markers CD63 and TSG101 as well as high level of TGF-β1. In MC38 cells, I-Evs were able to decrease the gene expression of IL-6, TNF-α, IL-1β, and IL-22 induced by *C. difficile* TcdB, but to increase both the gene expression and protein levels of TGF-β1. I-Evs treatment via intraperitoneal administration alleviates *C. difficile* TcdB-induced local colon inflammation in mice and increased their survival rate from 50% up to 80%. Furthermore, I-Evs induced an increase in the proportion of CD4^+^Foxp3^+^Tregs in vitro and in vivo through a TGF-β1-dependent mechanism by activating the TGF-β1 pathway and prompting phosphorylation of the downstream proteins Smad 2/3.

**Conclusion:**

For the first time, our study demonstrated that I-Evs originated from intestine epithelial cells can alleviate inflammation induced by *C. difficile* TcdB both in vitro and in vivo. Therefore, I-Evs might be potentially a novel endogenous candidate for effective treatment of CDI.

## Introduction

In recent decades, with the excessive application of broad-spectrum antibiotics, diseases related to intestinal flora disorders have precipitously increased. *Clostridioides difficile* (*C. difficile*) is one of the main pathogens leading to antibiotic-associated diarrhoea and hospital-acquired infections in the United States and other developed countries (Lessa et al. 2015). Toxin A (TcdA) and B (TcdB) are the major pathogenic factors leading to diarrhoea, pseudomembranous colitis, toxic megacolon, and other intestinal symptoms (Buonomo and Petri 2016). The mechanism lies in the inactivation in the host epithelial cells of proteins from the Rho family of GTPases-including Rho, Rac, or Cdc42 by glycosylation, and upregulation of a series of pro-inflammatory cytokines such as interleukin IL-1, IL-6, and TNF-α (Ng et al. 2010). Meanwhile, toxins recruit neutrophils and other inflammatory immune cells to induce intestinal mucosal cell apoptosis, necrosis, shedding, and increased permeability, triggering a widespread loss of intestinal barrier function, and initiating imbalance of flora and intestinal epithelial damage. According to the American Infectious Society, and the European Society of Clinical Microbiological Infections, in addition to other practical guidelines, oral metronidazole or vancomycin are the best methods to treat *Clostridioides difficile* infection (CDI) (Kociolek and Gerding 2016). In addition, some new narrow-spectrum antibiotics such as fidaxomicin (Louie et al. 2011) and rifaximin have little impact on the intestinal flora and reduce the risk of drug resistance. In recent years, a number of immune-based agents (Yang et al. 2014) have entered clinical trials, and however their efficacy needs to be further validated. Faecal microbiota transplantation (FMT) has been recognised in the United States as an optional treatment method to restore normal intestinal flora and prevent recurrent attacks. However, a meta-analysis of randomised clinical trials in 2019 showed that the cure rate of FMT was only 76.1%. Furthermore, there are still many unanswered questions about FMT, including the optimal timing, preparation methods, and the patients who are likely to benefit most from this procedure. As its standard protocol is relatively complicated and involves approval of ethical reviews, FMT has not yet been widely used in China.

Extracellular vesicles (Evs) are small vesicle-like substances secreted by cells, which possess various biological activities when released outside of the cell. They have a diameter ranging from approximately 30 nm to1 µm, and are generally classified into exosomes, microvesicles, and apoptotic bodies based on their size, biogenesis, and mechanism of secretion (Raposo and Stoorvogel 2013). It is difficult to determine the functional differences between these three types of Evs, due to the lack of specific markers with which to distinguish them. Although once thought to be cellular debris, Evs are now recognised as vital vehicles involved in the communication between cells. Research has confirmed that Evs contain a wide range of biologically active components, and their corresponding functions depend on the source tissue or cell type. Evs also exist in body fluids such as serum, alveolar lavage fluid, and breast milk, carrying messenger RNAs, microRNAs, and DNA (Colombo et al. 2014; Miyake et al. 2020); this suggests its potential applications as biomarkers for the diagnosis of diseases, as part of a liquid biopsy technology (Yang et al. 2020). Recently, it has been reported that Evs can be designed to function as effective carriers in the treatment of various diseases, including in the delivery of long non-coding RNAs (Babuta et al. 2019; Cao et al. 2019). In addition, Evs play a significant therapeutic role in regulating complex intracellular pathways in certain diseases, such as inflammatory bowel disease (IBD) (Wang et al. 2020; Wu et al. 2019), and osteoarthritis (Liu et al. 2019). Furthermore, it has been discovered that Evs derived from mesenchymal stem cells possess important immunomodulatory effects in areas such as neurodegenerative diseases, ageing, and inflammation (Williams et al. 2019; Boulestreau et al. 2020; Harrell et al. 2020). Previously, we have reported that CD8α^+^CD11c^+^ Evs derived from lungs reduce the allergic reaction of asthmatic mice through TGF-β1 and IL-10, thereby maintaining the immune balance of the respiratory tract (Wan et al. 1950). In the context of the recent outbreaks of COVID-19 around the world, mesenchymal stem cells and their Evs could be used as potential drug candidates for the treatment of severe cases, mainly through the induction of anti-inflammatory macrophages, regulatory *T* and *B* cells, and regulatory dendritic cells (Allan et al. 2020).

Strikingly, infection with TcdB-producing *C. difficile* strains alone, but not TcdA^+^B^−^ *C. difficile* strains, can cause severe CDI symptoms (Mileto et al. 2020). Our work presented here using purified *C. difficile* TcdB, together with cell lines and mice, confirmed that TcdB can induce expression of the inflammatory genes IL-6, TNF-α, IL-22, and IL-1β, and upregulation of TGF-β1 in vitro. Intestine epithelial cell-derived extracellular vesicles (I-Evs) rescue this phenomenon in vivo by inducing proliferation of regulatory *T* cells, dependent on TGF-β1 and the corresponding downstream molecules Smad2/3. Thus, we studied the role of I-Evs on inflammation induced by *C. difficile* TcdB and evaluated biological functions of I-Evs in alleviating pathological damage led by CDI in mice.

## Materials and methods

### Toxins, antibodies, and reagents

*C. difficile* TcdB was gifted from the Tao Liang research group (West Lake University, Hangzhou, China) (Shen et al. 2020). Primary antibodies against CD63 (ab213090), TGF-β1 (ab8227), GRP94 (ab238126), TSG101 (ab125011), and β-Actin (ab8227) were purchased from Abcam (Cambridge, MA, USA). PRMT1 (A33) (#2449), Smad2 (D43B4), Smad3 (C67H9), phospho-Smad2 (Ser465/Ser467) (E8F3R), and phospho-Smad3 (Ser423/425) were purchased from Cell Signalling Technology (Danvers, MA, USA), and the corresponding secondary antibodies were purchased from BBI (Shanghai, China). Fluorescent-labelled antibodies against CD4 (GK1.5) and Foxp3 (PCH101) were purchased from eBioscience (San Diego, CA, USA).

### Real-time fluorescence quantification PCR

The classic TRIzol (Gibco, USA) method was used to extract RNA, using a reverse transcription kit (TOYOBO) to acquire cDNA. Real-time, fluorescence quantification PCR (qRT-PCR) was performed in a Step One Plus Real Time PCR System (Roche) to detect gene expression. The mouse-specific primers used are shown in Appendix Table 1.

### Mouse and cell lines

The MC38 murine colon carcinoma cell line was purchased from Wuhan Fine Biotech Co., Ltd. (Wuhan, China). The cells were negative for mycoplasma as detected by fluorescence and culture methods. Human LOVO colon carcinoma cells were kindly provided by Jia Jing (Hangzhou Medical College, Hangzhou, China). Male C57BL/6 J mice (6–8 weeks old) were purchased from Shanghai Laboratory Animal Co., Ltd. (Shanghai, China). The mice were housed in a specific pathogen-free animal facility located in Laboratory Animal Centre in Hangzhou Medical College, and the approval number of Animal Care and Use was SYXK (Zhejiang Province) 2017–0013, and experimental protocols were approved by the Animal Care and Use Committee of Hangzhou Medical College, all animals were treated according to the guidelines for animal experimentation of Hangzhou Medical College in Hangzhou, China. The animal experiments were also performed in accordance with the ARRIVE (Animal Research: Reporting of In Vivo Experiments) guidelines (Boutron et al. 2020). The mice were sacrificed in 5 days after anesthetized with intraperitoneal injection chloral hydrate (375 mg/kg of body weight).

### Isolation and quantification of mouse I-Evs

Mouse large intestines were surgically extracted and ground in a sufficient volume of PBS. They were then digested with 1 mg/mL collagenase type II from *Clostridium histolyticum* (Gibco) for 2 h at 37 ˚C. The resulting suspension of intestinal tissue fragments was centrifuged at 400*g* for 10 min, and the supernatant carefully removed for further centrifugation at 10,000*g* for 30 min, to remove larger vesicles. The resulting supernatant was then filtered by a 0.22 µm screening and ultracentrifuged at 100,000*g* for 1 h. Crude pellets of I-Evs were washed in sterile PBS and centrifuged at the same speed for an additional 1 h. The harvested I-Evs were resuspended in PBS. A BCA assay was used to detect the concentration of I-Evs (ThermoFisher, Waltham, MA, USA).

### Electron microscopy scanning and nanoparticle tracking analysis

Suspensions of I-Evs were loaded onto a coated copper grid, and a drop of 2% phosphotungstic acid added as a negative staining method. The sample was then allowed to dry at room temperature and transferred to a transmission electron microscope (Hitachi H7650, Hitachi, China) to take pictures and record at a voltage of 80 kV. To detect size distribution, I-Evs were diluted with PBS, and 0.3 mL analysed by NanoSight Nano instruments (Malvern, UK).

### Western blot and flow cytometry analysis

For western blot analysis, 40 µg I-Evs or protein lysates extracted from intestinal tissues were separated by 12% sodium dodecyl sulphate–polyacrylamide gel electrophoresis (SDS-PAGE), and transferred to a polyvinylidene fluoride (PVDF) membrane (Millipore, Billerica, MA, USA). Membranes were blocked with 5% milk in phosphate buffered solution-Tween 20 (PBS-T) and incubated with the corresponding primary antibodies at 4 ℃ overnight. The next day, membranes were incubated with an HRP-coupled secondary antibody for 1 h at room temperature and scanned using a Canon 4500 imaging system (Shanghai, China). For flow cytometry analysis, cells were washed with cold PBS and incubated with a fluorescent antibody for 30 min at 4 ℃ in the dark. Cells were analysed by fluorescence-activated cell sorting (BD, Franklin Lakes, NJ, USA).

### CD4^+^Foxp3^+^Tregs induction assay

Murine CD4^+^ *T* cells were isolated with the EasySep Mouse CD4 + *T* Cell Isolation Kit (Stemcell), and labelled with an anti-CD62L antibody for flow cytometry. Magnetic sorting was then performed using the EasySep Mouse Biotin Positive Selection Kit (Stemcell). Cells were then incubated with 1 µl anti-CD3/CD28-coated beads and 200U/mL IL-2 for 72 h (2 × 10^5^ cells/well), with or without 50 µg/mL I-Evs in PBS. To block the TGF-β1 signal, 0.6 µg/mL TGF-β1 inhibitor was applied to cells (in vitro), or 15 µg/mL anti–TGF-β1–neutralising antibody was injected into mice (in vivo). The percentage of CD4^+^Foxp3^+^Tregs was analysed by flow cytometry.

### Induction and treatment of murine local colon inflammation induced by *C. difficile* TcdB

C57BL/6 J male mice were randomised into groups and given mixed antibiotics through their drinking water for 5 days. The antibiotic mixture consisted of gentamicin (0.035 mg/mL), kanamycin (0.4 mg/mL), colistin (850 U/mL), metronidazole (0.215 mg/mL), and vancomycin (0.045 mg/mL) (Sigma-Aldrich, St. Louis, MO, USA). The following day, mice were injected with clindamycin (10 mg/kg). Then, purified TcdB was surgical injected into local colon of mice (0.5 µg/kg); this was noted as day 0. Functional I-Evs (50 µg/100 µL in PBS) were administered through intraperitoneal injection after 5 h, and on day 1. After sacrificing the animals, the intestinal tissue in different groups was collected and prepared for H&E staining, and the tissue damage were scored by an inspector without prior knowledge of the experimental procedures, as described previously (Morteau et al. 2002).

### Statistical analysis

Data are presented as the mean ± SEM. Data were compared using a Student’s *t*-test with GraphPad Prism 8 (San Diego, CA, USA). *P* < 0.05 was considered statistically significant.

## Results

### Isolation and identification of intestine epithelial cell-derived extracellular vesicles

**Fig. 1:**
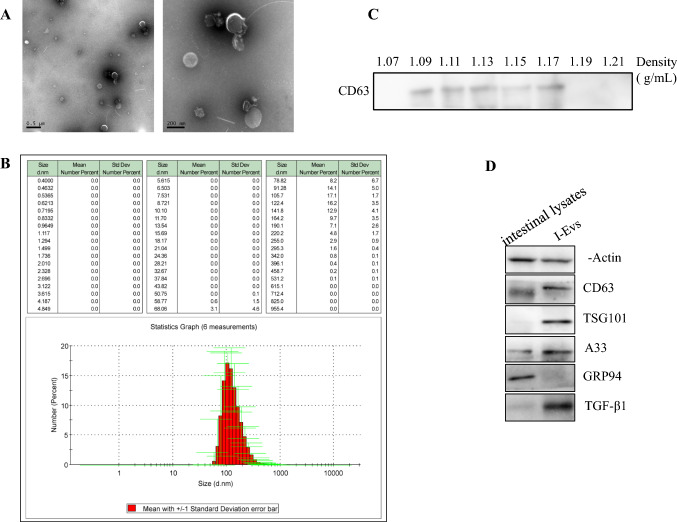
Identification of intestine epithelial cell-derived Evs (I-Evs). Extracellular vesicles were isolated from murine intestinal tissues and digested by standard procedures. **A**, **B** The morphology and diameter of I-Evs were analysed by electron microscopy and Nanoparticle tracking analysis. **C** 200 μg I-Evs were placed onto different concentrations of sucrose solution, and analysed by western blot with an anti-CD63 antibody. **D** 40 µg of intestinal lysates and I-Evs were separated by SDS-PAGE and transferred to a PVDF membrane. β-Actin, CD63, TSG101, A33, GRP94, and TGF-β1 were detected using antibodies. All data were verified by three independent experiments

The morphology of the purified I-Evs was visualised by transmission electron microscopy combined with Nanoparticle tracking analysis. The isolated I-Evs had a mean diameter of 100–200 nm (Fig. 1A, B). To further explore the I-Evs, sucrose density gradient centrifugation was used to detect the density range of I-Evs, which was 1.09–1.17 g/mL (Fig. 1C). Western blotting identified that I-Evs were positive for universal surface markers of extracellular vesicles, including CD63 and TSG101, and the intestinal epithelial cell-specific protein A33, but negative for GRP94 (Fig. 1D). In addition, high levels of TGF-β1 were present in I-Evs, implying a role in immunoregulation. The above results showed that I-Evs were successfully obtained.

### I-Evs attenuated the downregulation of TGF-β1 induced by purified *C. difficile* TcdB in vitro

**Fig. 2:**
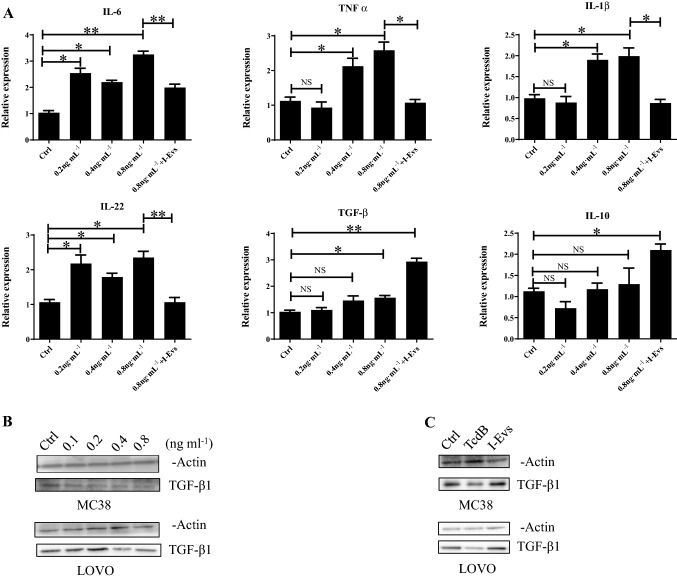
I-Evs attenuated the downregulation of TGF-β1 induced by purified *C. difficile* TcdB in vitro. **A** MC38 cells were exposed to different concentrations of TcdB (0.2 ng/mL, 0.4 ng/mL, or 0.8 ng/mL) for 5 h, or simultaneously treated with 50 µg I-Evs. Real-time PCR was used to detect gene expression levels of IL-6, TNF-α, IL-1β, IL-22, TGF-β1, and IL-10. **B** MC38 and LOVO cells were stimulated with *C. difficile* TcdB (0.1 ng/mL, 0.2 ng/mL, 0.4 ng/mL, or 0.8 ng/mL), before cell lysates were analysed by western blot. **C** Similarly, cells were treated with *C. difficile* TcdB (0.8 ng/mL) and I-Evs, the TGF-β1 protein levels analysed by western blot. All data were verified by three independent experiments. *P* values were calculated by one-way analysis of variance (ANOVA), versus control conditions (*P < 0.05, **P < 0.01, ***P < 0.001, *NS* not significant)

Real-time PCR results showed that in cultured MC38 cells treated with *C. difficile* TcdB or I-Evs, compared to the control group, the expression of pro-inflammatory genes (IL-6, TNF-α, IL-1β, and IL-22) was increased in the 0.4 ng/mL *C. difficile* TcdB group, but significantly decreased in the 0.8 ng/mL I-Evs group. In contrast, the expression of the anti-inflammatory genes TGF-β1 and IL-10 was significantly increased in the I-Evs group compared to the TcdB groups (Fig. 2A). Western blotting showed that protein levels of the immunosuppressive cytokine TGF-β1 were decreased both in MC38 murine colon carcinoma cells and LOVO human colon carcinoma cells after stimulated by *C. difficile* TcdB with concentration of 0.1 ng/mL, 0.2 ng/mL, 0.4 ng/mL, or 0.8 ng/mL, respectively (Fig. 2B). This reduction could be rescued by I-Evs treatment when TcdB concentration was 0.4 ng/mL (Fig. 2C). Altogether, these results indicate that the I-Evs containing TGF-β1 had anti-inflammatory effects in vitro.

### I-Evs alleviate *C. difficile* TcdB-induced local colon inflammation in mice

**Fig. 3:**
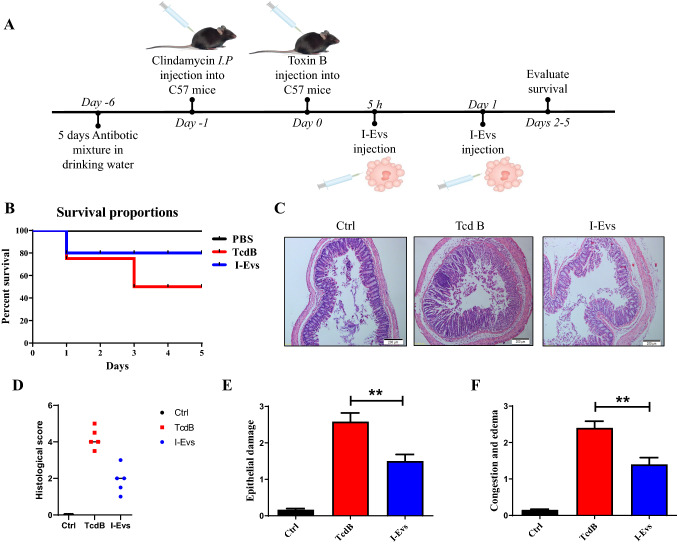
I-Evs alleviate murine *C. difficile* TcdB-induced local colon inflammation. **A** C57BL/6 J mice received antibiotic mixture for 5 days through their drinking water. The following day, mice were injected with clindamycin (10 mg/kg), followed by *C. difficile* TcdB (0.5 µg/kg) via surgical injection. This was noted as day 0. Functional I-Evs (50 µg/100 µL in PBS) were injected after 5 h and on day 1. **B** The survival rate of mice. **C**, **D** Intestinal tissue was collected and prepared for H&E staining and histological score. Epithelial damage **E** and congestion **F** were scored as histopathological severity. Images are representative of results from five animals, at the indicated time points after the TcdB challenge. All data were verified by three independent experiments. Values represent the mean ± SEM (*n* = 5 animals) versus control animals (*P < 0.05, **P < 0.01, ***P < 0.001, *NS* not significant)

Intestinal epithelial damage caused by *C. difficile* TcdB, generally confined to the local intestine, is a severe inflammatory intestinal lesion. We sought to explore whether I-Evs can be applied in this condition as a type of anti-inflammatory immunotherapy. I-Evs contain more TGF-β1 than intestinal lysates as determined by western blot, which indicates a likely strong immunosuppressive effect. Therefore, we established a murine local colon infection model to investigate the treatment effect of I-Evs (Fig. 3A). As shown in (Fig. 3B), the survival rate of mice after *C. difficile* TcdB injection was only 50%, while I-Evs treatment increased the survival rate of mice up to 80%. The intestinal tissues displayed marked leukocyte infiltration and sections of glandular structure damage caused by *C. difficile* TcdB; As expected, histopathological analysis showed only slight leukocyte infiltration and epithelial cell damage after application of I-Evs (Fig. 3C, D). Moreover, intestinal epithelial damage, congestion and mucosal oedema were significantly increased in the *C. difficile* TcdB mice when compared with the control mice (Fig. 3E, F). However, less intestinal damage and limited leukocyte infiltration were observed when mice were treated with I-Evs (Fig. 3E, F). These findings implied that I-Evs attenuated pathological changes occurring as a result of *C. difficile* TcdB-induced inflammation, thereby protecting mice from local colon inflammation.

### Induction of regulatory *T* cells by I-Evs alleviated infection caused by *C. difficile* TcdB through a TGF-β1-dependent mechanism

**Fig. 4:**
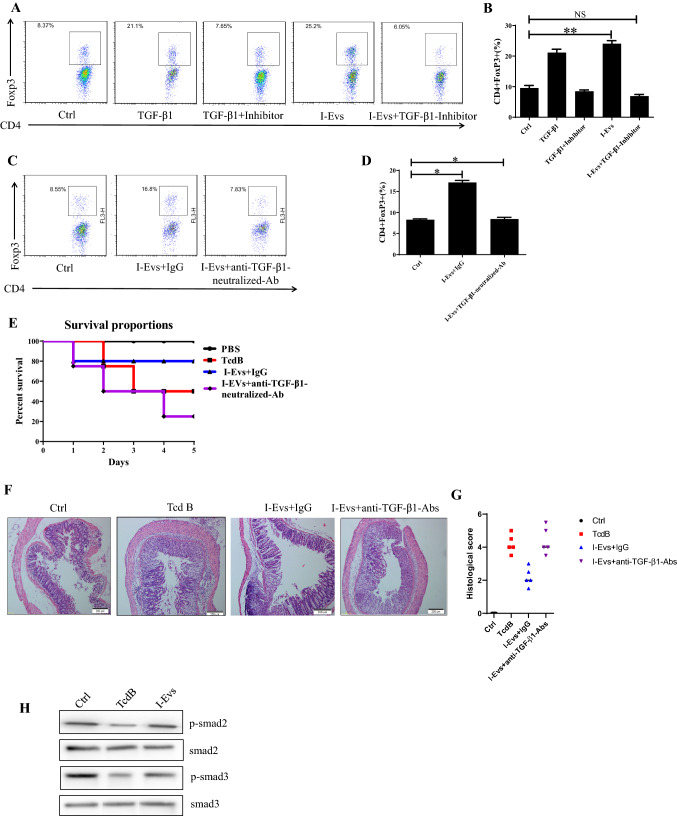
Induction of regulatory *T* cells by I-Evs alleviated local colon infection caused by *C. difficile* TcdB through a TGF-β1-dependent mechanism. **A** A lymphocyte suspension was obtained by grinding and filtering the spleen and lymph nodes of native C57 mice. Naïve CD4^+^ *T* cells were magnetically separated with the EasySep Mouse CD4^+^ *T* Cell Isolation Kit, and incubated with 200U/mL IL-2 and 1 µL anti-CD3/CD28-coated beads for 72 h (2 × 10^5^ cells/well), and separately treated with 3 ng/mL TGF-β1, 0.6 µg/mL TGF-β1 inhibitor, or 50 µg/mL I-Evs in PBS. **B** Statistical analysis of (**A**) (*n* = 9). **C** To block the TGF-β1 signal in vivo, 15 µg/mL anti-TGF-β1-neutralising antibodies were injected into mice and I-Evs were transfused three days later. The percentage of CD4^+^Foxp3^+^Tregs was analysed by flow cytometry. **D** Statistical analysis of (**C**) (*n* = 9). **E** The survival rate of mice. **F**, **G** The infiltration of neutrophils and destruction of intestinal cells and histological score. **H** Western blot analysis of Smad2/3, and phosphorylated Smad2/3, in MC38 cells stimulated with *C. difficile* TcdB. All data were verified by three independent experiments. *P* values were calculated by one-way analysis of variance (ANOVA), versus control animals (*P < 0.05, **P < 0.01, ***P < 0.001, *NS* not significant)

A previous study showed that EpCAM-dependent I-Evs alleviated IBD by inducing regulatory *T* cells (Jiang et al. 2016). I-Evs induced an increase in the proportion of CD4^+^Foxp3^+^Tregs in vitro and in vivo (Fig. 4A–D); these immunoregulatory cells exhibit immunosuppressive effects in the development of disease. When the activity of TGF-β1, a potent immunosuppressive cytokine, was blocked (using the protocol described in the Materials and Methods), I-Evs immediately lost the ability to induce CD4^+^Foxp3^+^Tregs in the spleen. Concurrently, I-Evs were not able to increase the survival rate of mice, and the improvement of pathological effects previously seen was also undetectable (Fig. 4E, F, G). Together, these results suggest that immunosuppressive regulatory *T* cells induced by I-Evs attenuated *C. difficile* TcdB- induced local colon inflammation in a mechanism dependent on TGF-β1. Smad2/3 are the main downstream proteins involved in the TGF-β1 signalling pathway. The phosphorylation levels of Smad2/3 were decreased after stimulation with *C. difficile* TcdB, although protein levels of Smad2/3 remained the same; treatment with I-Evs promoted phosphorylation of Smad2/3, and thereby upregulation of TGF-β1 (Fig. 4H). These results suggest that Smad2/3 is inhibited by *C. difficile* TcdB, leading to the downregulation of TGF-β1 expression. Conversely, I-Evs with high expression of TGF-β1 activate Smad2/3 and contribute to the upregulation of TGF-β1, thereby alleviating *C. difficile* TcdB-induced local colon inflammation in mice.

## Discussion

A common clinical symptom of CDI is local colon infection, which may arise due to intestinal perforation after either infection or intestinal surgery, particularly in high-risk populations, such as patients with IBD; respiratory insufficiency; heart and renal failure; ages over 60 years; and several other underlying diseases. The majority of CDI can be treated with metronidazole and fidaxomicin, in addition to other antibiotics. Surgical removal of necrotic intestinal tissue can reduce mortality rates with severe explosive colitis. Nevertheless, postoperative bleeding, and intestinal stenosis and obstruction, are extremely distressing to the patient. Prevention, management, and non-surgical treatment are the fundamental principles of CDI. However, the most severe toxic colitis cases are unable to benefit from drugs and surgery, and there is an urgent need to establish an effective treatment programme based on immunotherapy. Evs participate in a variety of physiological and pathological processes, including neurological disorders (Chung et al. 2020), osteoarthritis (Ni et al. 2020), infection (Kumar et al. 2020), and tumours (Scavo et al. 2020). Evs have been proven to be involved in immune regulation and antigen presentation, and our research group demonstrated that Evs derived from intestinal epithelial cells alleviate IBD in mice by inhibiting dendritic cell activation and inducing Tregs (Jiang et al. 2016). In this study, I-Evs isolated from the intestine, with mean diameters of 100–200 nm as detected by electron microscopy scanning and Nanoparticle tracking analysis, expressed the characteristic protein markers of Evs, CD63 and TSG101. Enrichment of the immunosuppressive cytokine TGF-β1 in I-Evs inspired us to hypothesise an immunomodulatory function for I-Evs. A recent study verified that Evs derived from human mesenchymal stem cells can relieve colitis by reducing pro-inflammatory responses and increasing anti-inflammatory responses (Ocansey et al. 2020).

As is well-established, colitis caused by *C. difficile* relies on a series of virulence factors, including toxins, which initially target intestinal epithelial cells and subsequently destroys the intestinal membrane integrity. Hosts exposed to intestinal microorganisms trigger immune inflammatory responses. The dominance of either TcdA or TcdB was still controversial in this research field, despite a multi-laboratory follow-up research study pronouncing that TcdB acts as a critical toxin in colonic epithelial injury and mortality in vivo, whereas TcdA caused inflammation in mice to a small extent (Carter et al. 2015). In the work presented here, TcdB-induced increased gene expression of IL-6, TNF-α, IL-1β, and IL-22. I-Evs were able to rescue this phenomenon, and interestingly, TGF-β1 and IL-10 gene expression actually increased upon co-incubation with I-Evs. Moreover, I-Evs could reverse the decrease of TGF-β1 stimulated by *C. difficile* TcdB, as detected by western blotting of MC38 and LOVO lysates. We also report for the first time that I-Evs can improve survival of mice with local colon inflammation induced by *C. difficile* TcdB. The mechanism lies in the induction of CD4^+^Foxp3^+^Tregs, which play an important role in maintaining immune tolerance and homeostasis; the decline or dysfunction of Tregs has previously been shown to increase intestinal inflammation in IBD mice (Yamada et al. 2016). Similarly, CD4^+^CD25^+^ Treg cells transferred into hosts ameliorated colitis symptoms.

Besides TGF-β1, IL-10 is also known to be an important immunosuppressive cytokine, but we could barely detect the presence of IL-10 in I-Evs obtained in this study. Whether IL-10 still performs an important function is unknown. Furthermore, pro-inflammatory cytokines were undetectable following stimulation with *C. difficile* TcdB. Indeed, we tried various schemes to optimise the experimental conditions, but failed to detect positive signals. Therefore, we speculate that the effect of *C. difficile* TcdB on cells in vitro was different from that in vivo. As for the animal challenge experiment, to induce chronic inflammatory intestinal infection, five antibiotic mixtures were fed to mice, in addition to intraperitoneal injection with clindamycin and local injection with *C. difficile* TcdB. Intraperitoneal administration of I-Evs improved both the survival of mice and intestinal tissue pathological scores significantly.

## Conclusions

For the first time, we demonstrated that I-Evs can alleviate inflammation induced by *C. difficile* TcdB both in vitro and in vivo, and protect against pathological lesions in the animal intestine using a mouse model. Moreover, our data indicated that I-Evs could activate the TGF-β1 pathway and the downstream proteins Smad 2/3, to alleviate local colon inflammation induced by *C. difficile* TcdB. Thus, I-Evs may provide as a novel endogenous candidate for treatment of *C. difficile* infections.

## Appendix

See Appendix Table 1

**Table 1:** Primer sequence for real-time PCR

Gene name	F&R	Primer sequence (5ʹ to 3ʹ)
β-actin	Forward	CACAGCTGAGAGGGAAATCGT
	Reverse	GCCATCTCCTGCTCGAAGTCTA
IL-6	Forward	AGTTGCCTTCTTGGGACTGA
	Reverse	TCCACGATTTCCCAGAGAAC
TNF-α	Forward	CTGGGACAGTGACCTGGACT
	Reverse	GCACCTCAGGGAAGAGTCTG
IL-1β	Forward	CCAAAAGATGAAGGGCTGCT
	Reverse	ACAGAGGATGGGCTCTTCT
IL-22	Forward	ATGAGTTTTTCCCTTATGGGGAC
	Reverse	GCTGGAAGTTGGACACCTCAA
TGF-β	Forward	CACCGGAGAGCCCTGGATA
	Reverse	GCCGCACACAGCAGTTCTT
IL-10	Forward	GGTTGCCAAGCCTTATCGGA
	Reverse	ACCTGCTCCACTGCCTTGCT
